# Possibility of Using Waste Materials as Substitutes for Gravel or Water in Concrete Mix

**DOI:** 10.3390/ma16051810

**Published:** 2023-02-22

**Authors:** Arkadiusz Bieszczad, Ernest Popardowski, Weronika Lubińska, Maciej Gliniak, Grzegorz Nawalany, Paweł Sokołowski

**Affiliations:** 1 Department of Bioprocess Engineering, Energy and Automation, Faculty of Production and Power Engineering, University of Agriculture in Krakow, Mickiewicza Av. 21, 31-120 Krakow, Poland; 2 Department of Machinery Exploitation, Ergonomics and Production Processes, Faculty of Production and Power Engineering, University of Agriculture in Krakow, Mickiewicza Av. 21, 31-120 Krakow, Poland; 3 Department of Rural Building, Faculty of Environmental Engineering and Land Surveying, University of Agriculture in Krakow, Mickiewicza Av. 21, 31-120 Krakow, Poland

**Keywords:** sewage sludge, solidification, concrete, rubber granulate, flexural strength, compressive strength

## Abstract

Analyzing the global waste management sector, we can see that some waste, due to its specificity, is a major challenge when it comes to its management. This group includes rubber waste and sewage sludge. Both items pose a major threat to the environment and human health. The remedy for this problem may be the solidification process, in which the presented wastes are used as substrates in the production of concrete. The aim of this work was to determine the effect of waste addition to cement in the form of an active additive (sewage sludge) and a passive additive (rubber granulate). An unusual approach to sewage sludge was used, which was introduced as a substitute for water, and not, as in most works, sewage sludge ash. In the case of the second waste, commonly used tire granules were replaced with rubber particles resulting from the fragmentation of conveyor belts. A wide range of the share of additives in the cement mortar was analyzed. The results for the rubber granulate were consistent with numerous publications. For the addition in the form of hydrated sewage sludge, the deterioration of the mechanical properties of concrete was demonstrated. It was found that the flexural strength of the concrete in which water was replaced with hydrated sewage sludge was lower than that of the sample without the addition of sludge. The compressive strength of concrete with the addition of rubber granules was higher than the control sample and did not significantly depend on the amount of granulate used.

## 1. Introduction

Solidification/stabilization is a more common modification technique used to immobilize contaminants, including radioactive and toxic ones [1,2], chemical fixation and the physical encapsulation of hazardous components [3,4] contained in wastes. Fly ash from the incineration of municipal solid waste is by far the most common type of waste subjected to this process [5,6]. However, other materials are also solidified, including: grounddry contaminated plants [7], ground granulated blast furnace slag, fly ash [8], polymeric wastes [9], nanomaterials [10,11] or food and plant residues as alternative fuels [12,13].

Cement-based stabilization/solidification has advantages over other solidification methods such as low cost, ease of use, quick waste processing and high durability [14]. For this purpose, the so-called Portland cement is used [15,16]. Waste substance can be used as a filler (passive additive) or as an alternative substitute for a binder component (active additive) in concrete production technology.

One of the wastes whose disposal on a global scale is currently a major challenge is sewage sludge. The joint discharge of municipal and industrial sewage causes sewage sludge to be exposed to contamination with heavy metal compounds, disease-causing bacteria, parasite eggs, fungi and other substances that pose a threat to the environment [17]. Sewage sludge can be subjected to cement-based stabilization/solidification; however, it should be remembered that an increase in the proportion of sewage sludge in concrete causes an exothermic reaction. The increasing heat that is generated during hardening processes often causes negative and often irreversible damage such as cracking and structural degradation of the concrete [18].

The second type of waste, in the case of which new ways of reuse or management are still sought, are used tires. After their fragmentation into the form of granules, it is possible to use tires as a passive additive to concrete. Floors constructed with cement and rubber granulate ingredients provide effective slip aid, assisting performance and using a design action different from conventional bitumen products [19,20]. At the same time, as indicated by Sofi [21], the addition of rubber as a substitute for the mineral fraction in concrete mix contributes to a decrease in the compressive strength and elasticity of the sample with an increase in the percentage of rubber. The cement grout containing rubber is much softer than without rubber. This phenomenon causes the rapid development of cracks around the rubber particles, thus reducing the strength of the finished concrete.

Analyzing scientific reports on the implementation of various types of admixtures in the production of concrete, it can be concluded that this form of waste management can solve many problems. The percentages of both mineral and biological additives can have a positive impact on the mechanical and chemical parameters of the final product. Jin P et al. tested the use of bio-admixtures instead of traditional chemical admixtures in the process of green concrete preparation. The results revealed that carrot extract (CS) effectively inhibited the early hydration of cement and prolonged its setting time. The setting time of the pastes containing CS was 1.1–2.7 times that recorded for the control group [22]. Analyzing the eco-admix residues obtained from waste incineration, we can conclude that their 15% replacement for cement allows it to obtain results similar to ordinary Portland cement [23,24]. Looking at the subject from a different angle, as another raw material in the production of concrete, waste paint can be used due to its similar composition to polymeric admixtures commonly used in the industry. It was found that a 12% addition of paint to the mixing water allows obtaining concrete with parameters similar to standard concrete [25]. It was also found that the use of this type of admixture allows not only the improvement of the basic properties of the construction composition, but also a reduction in water absorption [26]. For concrete enrichment, we can also go further. Epoxy plastic, a form of epoxy resin waste, can be mixed with concrete to enhance the thermal and compressive resistance and tensile strength [27]. The 5% addition of hydrogen peroxide and phosphogypsum led to positive changes in the final properties of the product. Porous concrete based on alkali-activated slag had compressive strength in the range of 2.12 to 7.95 MPa, and a density from 830 kg/m^3^ to 1142 kg/m^3^ [28,29]. Amin M. et al. tested the use of recycled waste glass in ultra-high-performance concrete. Replacing Portland cement with powdered glass resulted in a reduction in the compressive force of all samples after one day. However, in the long term (7, 28 and 91 days) it was noted that the compressive strength increases up to 20% replacement ratio [30]. Waste glass can be used as cementitious material (with sizes finer than 100 μm) or virgin aggregate in concrete, according to its size. Furthermore, the effect of the addition of glass on the overall density and modulus of elasticity is negligible [31,32]. However, the use of steel fiber-reinforced rubberized concrete allows for a significant improvement in the parameters of the beams created in this way. The experimental results show that reinforced beams made of similar strength Crumbed Rubber Concrete have similar ultimate flexural capacities regardless of rubber content [33]. However, other researchers drew the opposite conclusions, noting that in the case of reinforced beams, the admixture of both rubber granules and powdered glass contributes to a decrease in compressive strength. However, both noticed the need for further verification of the obtained results, noting problems with the agglomeration of admixtures in the volume of concrete [34,35]. The aim of this work was to determine the effect of two waste cement additives on cement’s physical and mechanical properties. 

The novelty of these studies is based on a different approach to the use of admixtures in concrete products. In the literature, there are positions analyzing the potential for the use of ashes generated in the processes of thermal transformation of sewage sludge or granulate from the grinding of used tires. Analyzing the topic of waste management, we found that there is a possibility to shorten the entire process of sewage sludge management. In this work, it was checked how the use of hydrated sludge as a substitute for mixing water will affect the final parameters of concrete. By using such technology, it would be possible to avoid the need for thermal processing of sludge and to use it in its raw form in the production of technical concrete. In this step, we noticed a novelty in the conducted research, because this type of approach is not commonly described in the literature and the examples appearing in articles do not provide clear information on the effectiveness of such admixtures. The second part of this work also assumes a slightly different approach to the subject. In opposition to other researchers, waste rubber from used tires was not used in the prepared concrete samples, but rubber granulate from the grinding of belt conveyors with a grain diameter of 0.5 to 2.5 mm was used. The rubber from which the conveyors are made has different parameters than that used in tires, and the amounts in which this waste is generated makes it necessary to conduct further work on the potential of its use. As part of the work carried out, it was checked how admixtures of sewage sludge in the amount of up to 100% and granulate up to 50% affect changes in the parameters of technical concrete.

## 2. Materials and Methods

### 2.1. Sewage Sludge

**Figure 1:**
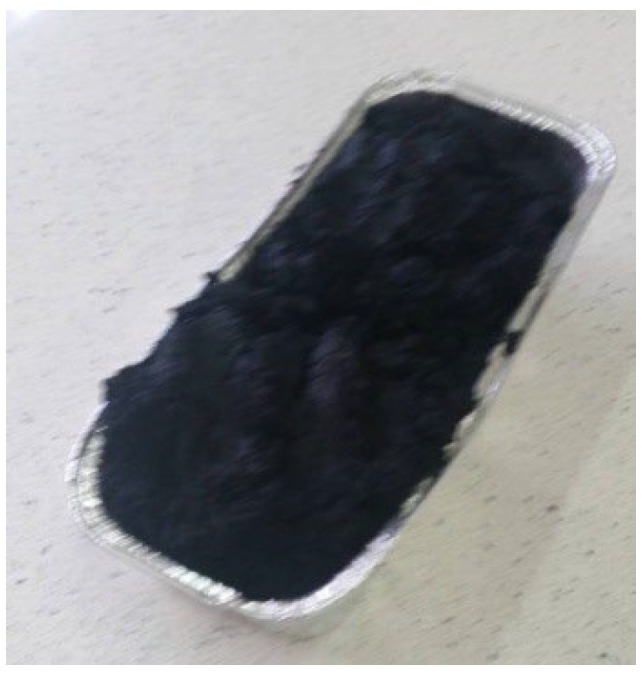
Sewage sludge used for research.

The hydrated sewage sludge (BioMed Ltd., Piekoszow, Poland) came from a municipal sewage treatment plant located in southern Poland. The sewage sludge (Figure 1) used was a mixture of primary and secondary biological sludge, with a moisture content of 84 ± 0.2%. Table 1 shows the basic properties of the sewage sludge.

### 2.2. Rubber Granulate

**Figure 2:**
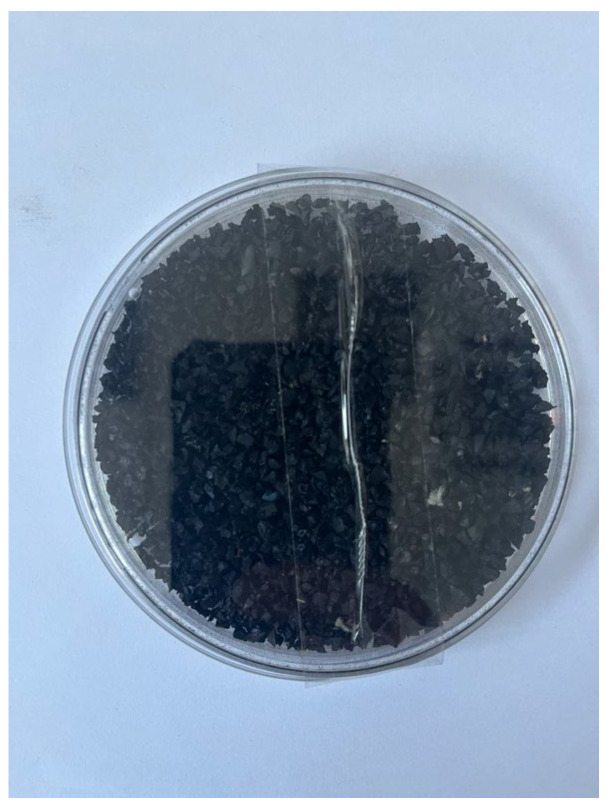
Rubber granulate used for research.

Rubber granulate (Figure 2) from the grinding process of belt conveyors, which is a waste based on various rubbers (mainly Styrene–Butadiene Rubber, (Unirubber Ltd., Węgliniec, Poland)), was used to prepare the samples. Table 2 shows the basic properties of the granulate.

### 2.3. Cement

The experiments used a dry concrete mortar class C16/20, according to the classification of the EN 206:2014-4 standard [36]. This means that the cement has a dry density of 2000 kg∙m^3^ to 2600 kg∙m^3^, and the compressive strength of the prepared cement for cylindrical samples should be at least 16 MPa, and for cubic samples at least 20 MPa.

### 2.4. Sample Preparation

The first series of samples consisted of a mixture of dry cement, water and sewage sludge. Subsequent samples contained 0%, 25%, 50%, 75% and 100% of hydrated sewage sludge used as a water substitute.

The second series of samples containing rubber granulate admixtures was based on the proportions of the reference mixture, taking into account additives representing the percentage replacement of aggregate in the mixture. Mixtures with a rubber pulp content of 0%, 5%, 10%, 20% and 50% were prepared. In order to optimize the mixing process and mortar preparation, all samples from the first and second series were prepared in a properly scaled form with particular emphasis on the proportions of individual substrates. 

The mortar was mixed with an electric mixer. The ready concrete mix was immediately transferred to two types of molds. The first one was a rectangular form with dimensions of 40 mm × 40 mm × 160 mm. The second were cylinders with a diameter of 40 mm and a height of 160 mm. The molds were attached to a shaker. After the application of the first layer, compaction took place (60 shakes), and the next layer was applied in excess and also compacted with 60 shakes. Excess mortar was removed with a simple scraping ruler. After forming, the samples were placed in a climatic chamber with constant conditions allowing free flow of air to each of the samples. After 24 h from the start of the molding process, the samples were removed from the mold. 

### 2.5. Tests

The prepared concrete was subjected to three tests. The first of them referred to the already withdrawn standard and allowed to determine the mass water absorption of concrete. For this purpose, the finished blocks were placed in a vessel with water at a temperature of 20.0 ± 1 °C in such a way as to allow water to be supplied directly to each sample. Samples were immersed gradually, every few hours, in such a way that complete immersion in water took place only after 24 h. The samples were then kept in water for another 48 h. After this time, weight gain measurements were performed. Mass water absorption is the ratio of the mass of water absorbed through the material to the weight of the dry material.

**Figure 3:**
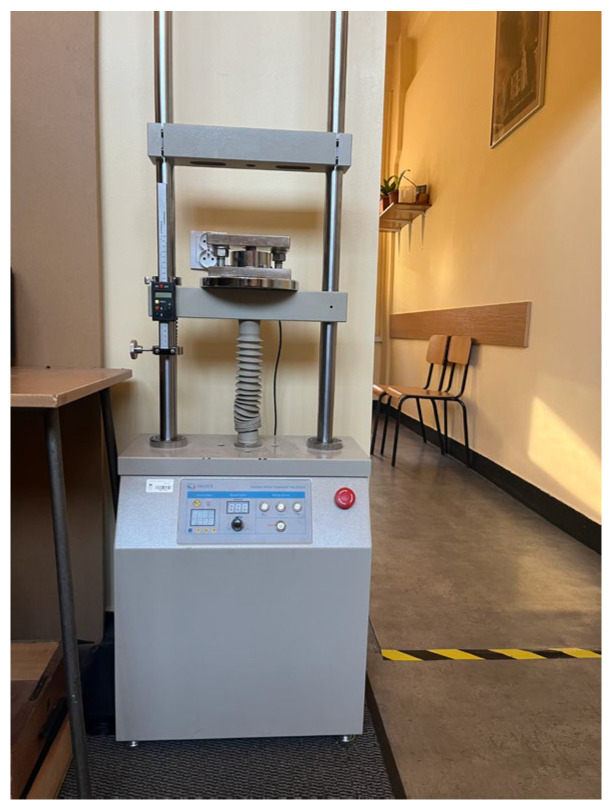
Sauter testing machine.

Strength tests were performed in accordance with the guidelines of EN 196-1:2016 [37]. Flexural strength was carried out on a SAUTER testing machine, (CR 30000-1Q1, Sauter, KERN&SOHN GmbH, Balingen, Germany (Figure 3). It should be noted that only cylindrical samples were selected for strength tests. The samples were placed on the supports with the lateral surface in the center of the measuring device disc and uniformly loaded with a force of 2400 ± 200 N·s ^−1^ until the sample was destroyed. The compressive strength test was carried out using the same device and, in accordance with the guidelines of the standard, half of the samples obtained during the bending strength test were used. The samples were broken without damaging the structure. The tested object was placed between the compression plates in the longitudinal direction. The load increment was set, as before, to a force of 2400 ± 200 N·s ^−1^.

In order to confirm or reject the hypothesis about the normal distribution of the examined parameter, the results of the research were subjected to the Kolmogorov–Smirnov test. Afterwards, selected basic descriptive statistics were calculated [38,39,40] as significant due to the described phenomena.

## 3. Results

### 3.1. Concrete with the Addition of Sewage Sludge

**Figure 4:**
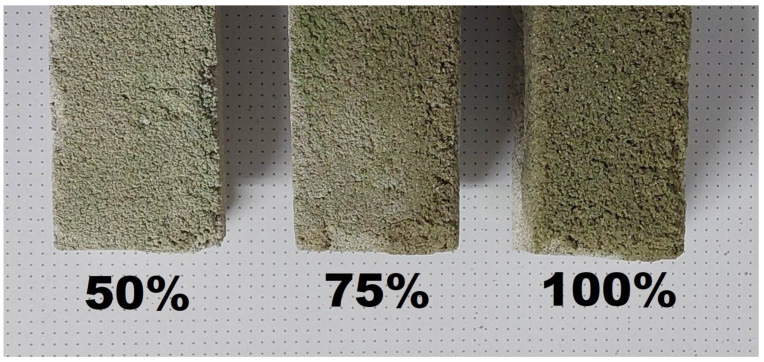
Examples of concrete samples with sewage sludge addition.

**Figure 5:**
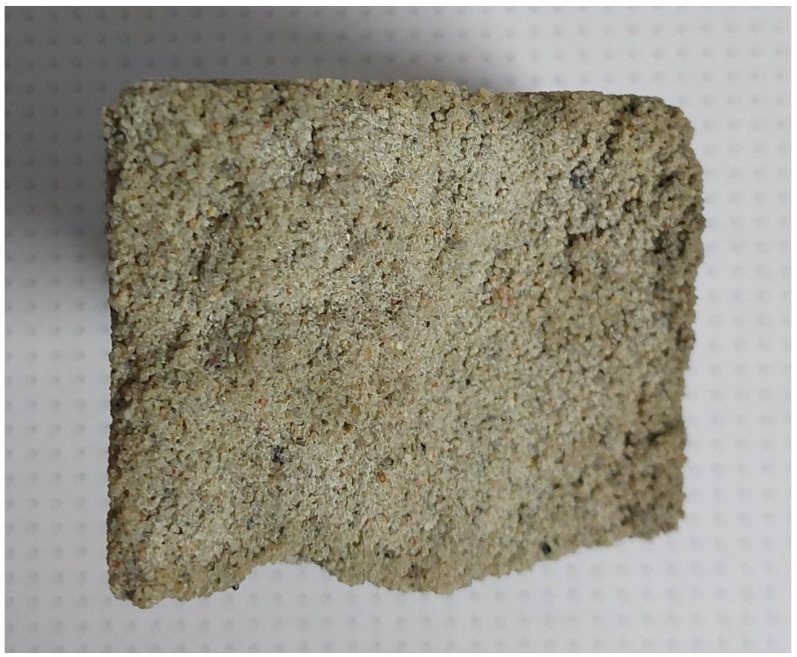
Examples of V2 concrete samples after flexural test.

For a given series of tests, hydrated sewage sludge was used as a water substitute. This means that the total amount of liquid needed to prepare the concrete was constant, but the sludge to water ratio varied. Five variants were prepared according to the following notation: V0 (control, 100% water); V1 (75% water, 25% sewage sludge); V2 (50% water, 50% sewage sludge); V3 (25% water, 75% sewage sludge); V4 (100% sewage sludge)—Figure 4 and Figure 5. 

**Figure 6:**
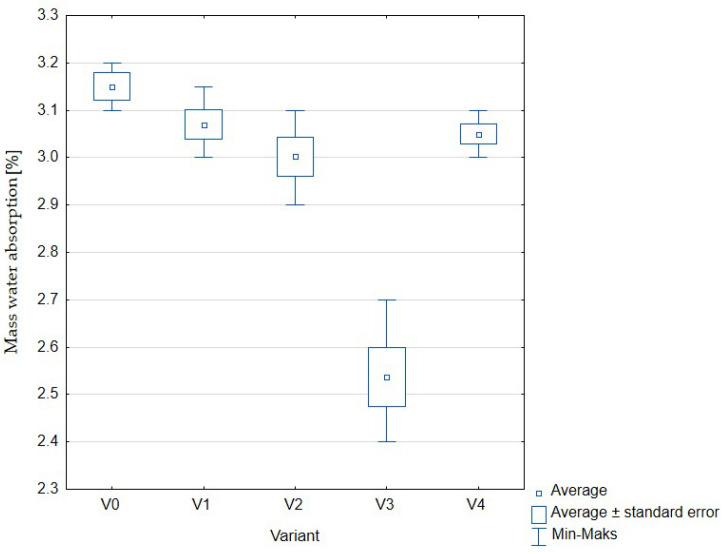
Mass water absorption of concrete with the addition of sewage sludge: V0 (control, 100% water); V1 (75% water, 25% sewage sludge); V2 (50% water, 50% sewage sludge); V3 (25% water, 75% sewage sludge); V4 (100% sewage sludge).

The results obtained for the measurements of mass water absorption, broken down into the variants described above, are presented in Figure 6, Table 3.

For all variants, except for the one in which the ratio of hydrated sewage sludge to water was 75%:25% (variant V3), the obtained results were not statistically significant. However, it should be noted that concrete soaking is a negative phenomenon, and thus the obtained results should be as low as possible. Mass water absorption in the tested variants did not exceed 4%.

**Figure 7:**
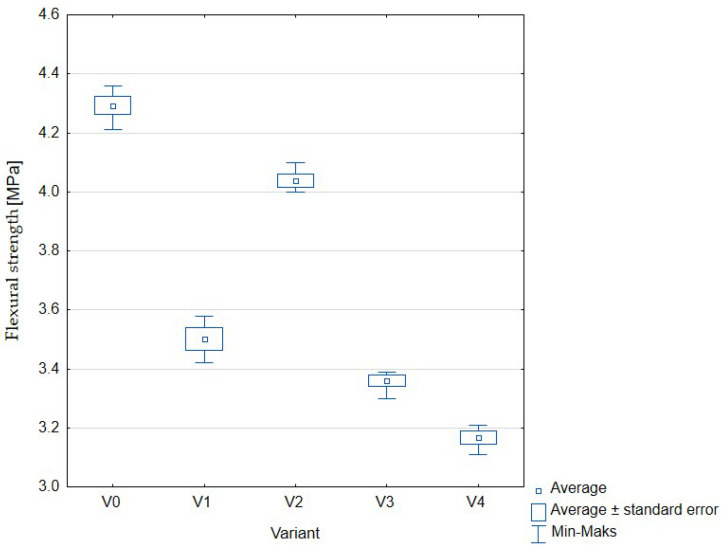
Flexural strength of concrete with the addition of sewage sludge: V0 (control, 100% water); V1 (75% water, 25% sewage sludge); V2 (50% water, 50% sewage sludge); V3 (25% water, 75% sewage sludge); V4 (100% sewage sludge).

Figure 7 (Table 4) shows the flexural strength of individual samples expressed in MPa. This parameter, in contrast to compression, is not usually defined in relation to concrete. However, it seems essential from the point of view of some technical applications, for example: concrete floors [26].

The obtained results were statistically significant and average values ranged from 4.27 to 3.20 MPa. It should be noted that in all variants, the differences for subsequent repetitions were small, as shown by the lines showing the minimum and maximum values for each result. The additions of hydrated sewage sludge had a negative effect on the flexural strength of the prepared concrete. Increasing the share of sludge as a water substitute resulted in a decrease in the obtained values, although for one variant, in which water and sludge accounted for 50% each, the obtained result was close to the value obtained in the case of the control sample (variant V1).

**Figure 8:**
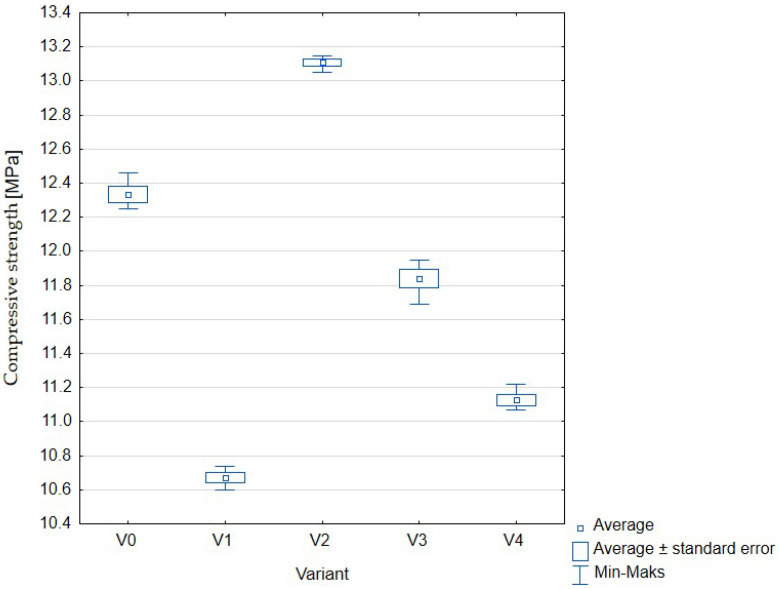
Compressive strength of concrete with the addition of sewage sludge: V0 (control, 100% water); V1 (75% water, 25% sewage sludge); V2 (50% water, 50% sewage sludge); V3 (25% water, 75% sewage sludge); V4 (100% sewage sludge).

One of the most important properties of concrete, which is also the basis for determining its class, is compressive strength (Figure 8, Table 5). According to the description presented in the methodology, using, as in this experiment, cylindrical samples, the obtained results should be characterized by a value of at least 16 MPa.

The obtained results were reproducible and statistically significant. Despite this, even for the control sample, the declared strength of 16 MPa or more was not obtained. When analyzing only those variants in which sewage sludge was added (V1–V4), similarly to the flexural strength test, the use of a sewage sludge to water ratio of 1:1 brought the most satisfactory results. This means that for variant V2, the compressive strength was the highest and even exceeded the value obtained for the control test (V1). The use of other proportions resulted in a decrease in the tested parameter in relation to the sample without the addition of sewage sludge.

### 3.2. Concrete with the Addition of Rubber Granulates

**Figure 9:**
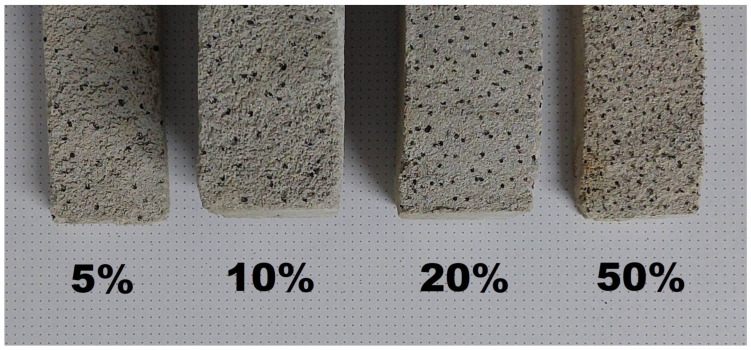
Examples of concrete samples with rubber addition.

**Figure 10:**
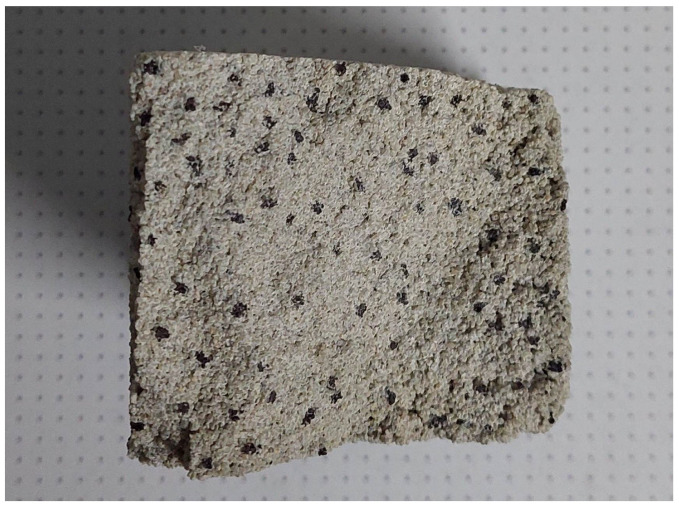
Examples of W3 concrete samples after flexural test.

In a given series of tests, cement mortar was mixed with water, but an additive in the form of rubber granulates was used. As in the case of the first series of tests, five variants were prepared in which the mass share of rubber in the entire concrete mix was changed. In order to simplify identification and clearly distinguish variants within both groups, the designations W0–W4 have been used for a given series. The subsequent variants were as follows (Figure 9 and Figure 10): W0 (control, 0% rubber additive); W1 (5% rubber additive); W2 (10% rubber additive); W3 (20% rubber additive); W4 (50% rubber additive).

**Figure 11:**
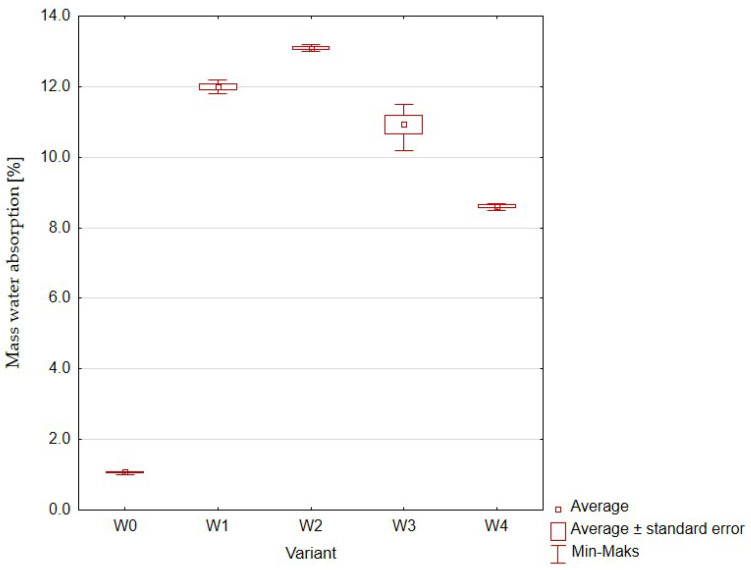
Mass water absorption of concrete with the addition of rubber granulates: W0 (control, 0% rubber additive); W1 (5% rubber additive); W2 (10% rubber additive); W3 (20% rubber additive); W4 (50% rubber additive).

The first of the analyzed parameters was mass water absorption (Figure 11, Table 6).

The absorbency of samples containing rubber granulate increased several times compared to the control sample, i.e., a mixture of cement and water without any additives. The highest average mass absorption of 12.75% was recorded for the sample containing 10% rubber granulate addition. An increase in the share of admixtures above this value resulted in a decrease in the absorptivity of concrete. The hypothesis put forward before the research assumed that the use of rubber granules would contribute to increasing the porosity of the samples and, as a result of capillary phenomena between the rubber particles, the amount of water that would be absorbed by the samples would increase. Meanwhile, the obtained values (statistically significant differences between the variants) do not show the assumed trend.

**Figure 12:**
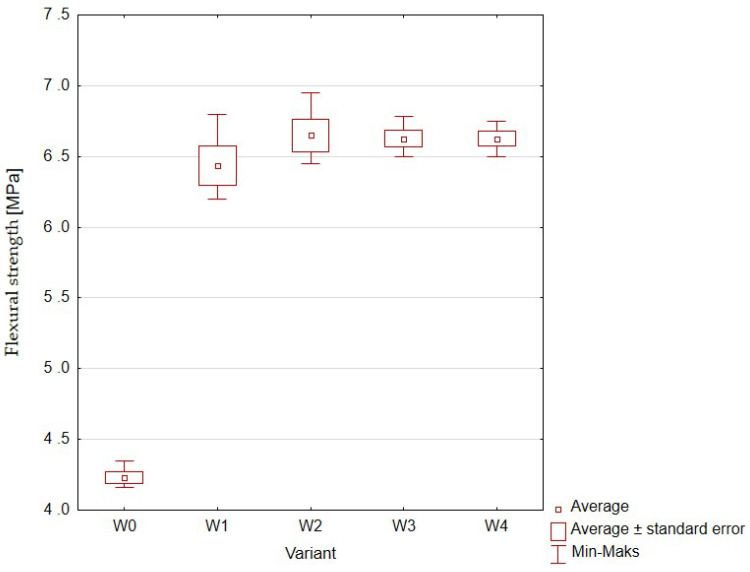
Flexural strength of concrete with the addition of rubber granulates: W0 (control, 0% rubber additive); W1 (5% rubber additive); W2 (10% rubber additive); W3 (20% rubber additive); W4 (50% rubber additive).

Flexural strengths of cylindrical concrete samples with different amounts of rubber addition are shown in Figure 12 and Table 7.

Although the values obtained for the variants from W1 to W4 were about 50% higher than the control sample, the differences between the samples with various additions of rubber granulate were not statistically significant, and the average values in all four variants oscillated in the range of 6.6–6.7%. This means that these differences were within the measurement error range for this method.

**Figure 13:**
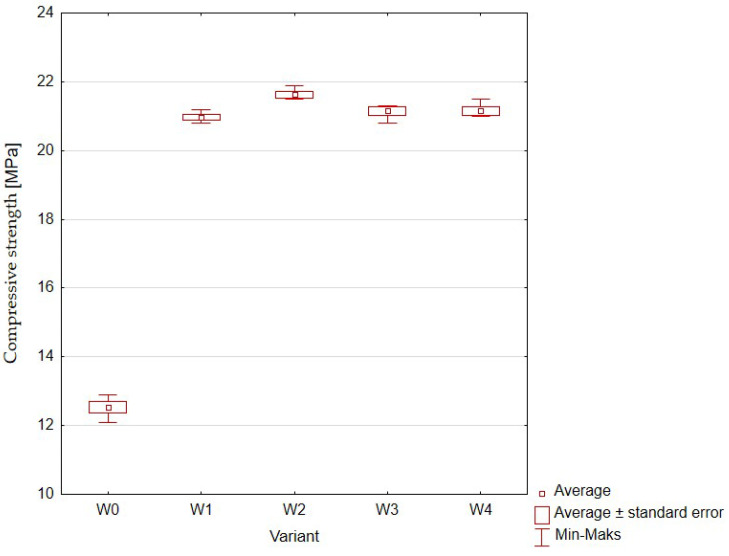
Compressive strength of concrete with the addition of rubber granulates: W0 (control, 0% rubber additive); W1 (5% rubber additive); W2 (10% rubber additive); W3 (20% rubber additive); W4 (50% rubber additive).

Figure 13 (Table 8) presents the compressive strength of concrete samples containing the addition of rubber granulate and for the sample without additives.

The results of the compressive strength test were characterized by high repeatability within each of the variants (there were few outliers). The control sample showed a value convergent with the control sample used in the first series of the experiment. The average values for the variants in which the rubber addition was used were very similar (between 21.0 and 21.6 MPa) and the values were larger by about 10 MPa compared to the sample without additives.

## 4. Discussion

Sewage sludge is an inevitable by-product of wastewater treatment. Depending on the concentration of pollutants in the sludge, various methods of their disposal can be used [41]. Numerous studies have indicated the potential possibility of using sewage sludge ash (SSA) as a substitute for cement or an additive to it [42,43,44,45]. However, it should be noted that even if some authors use the term sewage sludge in the titles of their works or abstracts, after a thorough analysis of the text, it turns out that they mean sewage sludge ash.

Cyr et al. [46] tested the compressive and flexural strength of mortars with 25% and 50% of cement replaced by SSA and found that sewage sludge ash causes reduction in strength compared to control mortars, but such reduction decreases over time. Lin et al. [47] found that smaller sewage sludge ash particles possess greater pozzolanic activity, resulting in a higher compressive strength of mortars. Chen et al. [48] reported satisfactory compressive strength of concrete prepared by using SSA to substitute 10% of cement and 2% of sand simultaneously. Ing et al. [49] also reported similar or greater strength values in mortars containing up to 10% sewage sludge ash in the binder. In turn, Rusănescu et al. [50] state the possibility of using cement with the addition of ash from sewage sludge as an additive in the cement industry. According to these authors, the compressive strength after 28 days is greater than or equal to 42.5 MPa.

Relatively few publications concern the effect of SSA addition on cement hydration. Dhir et al. [51] also stated in their book that most studies on the porosity of SSA-containing mortar or concrete involved various types of water absorption tests, but not an in-depth analysis of the microstructure. Moreover, as reported by Lynn et al. [52], although drying shrinkage is a parameter that controls the durability of ordinary concrete, there have been very few studies evaluating the effect of SSA on the bulk stability of mortars due to drying shrinkage and related mechanisms. In addition, inconsistent results were obtained regarding the workability of fresh SSA-containing mortars. 

The issue is different with the second-tested waste. The use of rubber granulate as an additive to cement is a commonly used technique [53,54,55,56]. It should be noted, however, that despite numerous studies on this subject, there are still issues for which no clear result has been achieved. An example is the penetration of concrete with the addition of rubber granulate by chloride ions, where some researchers found an increase in this permeability after adding rubber cement [57,58], and others showed a decrease in the subject penetration [59,60]. 

In the case of rubber addition, not only its type and the ratio of rubber to other concrete components, but also the particle size seems to be important. According to Karunarathna et al. [61], the larger the rubber particle size, the worse the mechanical properties of the cement mortar. Publications on the use (as in this work) of particles not exceeding 2.5 mm constitute a large percentage of published works [62,63,64,65]. However, as pointed out by Su et al. [63], the addition of small rubber particles increases water absorption, which was also proven in this work.

A number of experimental studies have shown that the addition of rubber granulate to concrete mixes in most cases reduces their compressive strength and flexural strength. Ismail and Hassan [66] indicated a decrease in compressive strength reaching nearly 60% in relation to pure concrete with the addition of 30% home-made granulate. Fernández-Ruiz et al. [67] showed that a 5% addition of rubber can cause a decrease in compressive strength of over 38%. Similar results were obtained by Mishra and Panda [68]; however, da Silva et al. [69] proved a more than 2% increase in the tested parameter with a 10% addition of rubber granulate. The last of the cited works is also one of the few in which the share of the additive in the form of rubber exceeding 30% of the composition of the mixture was analyzed.

In the case of flexural strength, there are studies showing both an increase in the strength of the cement with the addition of rubber relative to the base mix [70], and a decrease in the analyzed parameter [71].

## 5. Conclusions

Replacing water in concrete with hydrated sewage sludge leads to a decrease in mass water absorption. The most significant difference was achieved with a sludge to water ratio of 3:1. The flexural strength of the concrete in which water was replaced with hydrated sewage sludge was lower than that of the sample without the addition of sludge, with the smallest decrease in the analyzed variants being recorded for the variant in which sewage sludge and water accounted for 50% each. The compressive strength of concrete with the addition of hydrated sewage sludge reached lower parameters than the control sample, with one exception (regarding the bending strength), i.e., the sample with an equal ratio of water to sewage sludge. Similar to the bending strength, the compressive strength of concrete with the addition of rubber granules was higher than the control sample and did not significantly depend on the amount of granulate used.

The mass water absorption was significantly greater in the case of replacing the cement with rubber granulate than in the control mix. The highest of the recorded results concerned the 10% addition of rubber. In the cement with the addition of rubber granulate instead of traditional rubber particles, the flexural strength clearly increased, but no statistically significant differences were observed between the successive variants in which the share of rubber was gradually increased.

It should be noted that even the control samples did not reach the parameters set for concrete mortar class C16/20.

The presented research results do not give a clear answer on the impact of additives such as raw sewage sludge and rubber granulate from the processing of belt conveyors on the physical and mechanical parameters of concrete. On the basis of the research carried out, the need to carry out further work on the influence of the presented substrates is still noticeable. As recommendations for further work, it is envisaged to check whether sewage sludge from specific industrial sectors can have a more significant impact on the improvement of the analyzed parameters. In addition, the verification of the proportion of substrates in the mixtures, as well as the technology of mixing and compaction itself, is a wide field for further analysis. 

## Figures and Tables

**Table 1:** Properties of the sewage sludge.

Property	Value	Unit
Grain size	<0.05	mm
Granules below 0.5 mm	100.0	%
Mineral impurities	max. 1.0	%
Bulk density	993.0	g∙dm^−3^
Shape	amorphous	-

**Table 2:** Properties of the rubber granulate.

Property	Value	Unit
Grain size	0.5–2.5	mm
Granules below 0.5 mm	max. 1.0	%
Granules above 2.5 mm	max. 5.0	%
Textile impurities	max. 1.0	%
Bulk density	<550	g∙dm^−3^
Shape	cubical particles	-

**Table 3:** Mass water absorption of concrete with the addition of sewage sludge.

Sample	Subsample	Water Absorption [%]
V0	1	3.1
	2	3.2
	3	3.2
	4	3.1
V1	1	3.2
	2	3.1
	3	3.0
	4	3.1
V2	1	3.0
	2	3.1
	3	3.0
	4	2.9
V3	1	2.6
	2	2.4
	3	2.5
	4	2.7
V4	1	3.0
	2	3.1
	3	3.1
	4	3.1

**Table 4:** Flexural strength of concrete with the addition of sewage sludge.

Sample	Subsample	Flexural Strength [MPa]
V0	1	4.3
	2	4.4
	3	4.3
	4	4.3
V1	1	3.5
	2	3.5
	3	3.4
	4	3.5
V2	1	4.1
	2	4.1
	3	4.1
	4	4.0
V3	1	3.4
	2	3.3
	3	3.4
	4	3.4
V4	1	3.2
	2	3.2
	3	3.2
	4	3.2

**Table 5:** Comprehensive strength of concrete with the addition of sewage sludge.

Sample	Subsample	Comprehensive Strength [MPa]
V0	1	12.3
	2	12.4
	3	12.3
	4	12.5
V1	1	10.7
	2	10.6
	3	10.7
	4	10.7
V2	1	13.1
	2	13.2
	3	13.1
	4	13.1
V3	1	11.9
	2	11.9
	3	11.7
	4	11.8
V4	1	11.1
	2	11.2
	3	11.1
	4	11.1

**Table 6:** Mass water absorption of concrete with the addition of rubber granulates.

Sample	Subsample	Water Absorption [%]
W0	1	1.1
	2	1.0
	3	1.1
	4	1.1
W1	1	12.0
	2	11.9
	3	12.0
	4	12.2
W2	1	13.1
	2	13.1
	3	12.9
	4	13.0
W3	1	11.0
	2	11.1
	3	11.1
	4	11.0
W4	1	8.7
	2	8.5
	3	8.6
	4	8.7

**Table 7:** Flexural strength of concrete with the addition of rubber granulates.

Sample	Subsample	Flexural Strength [MPa]
W0	1	4.2
	2	4.4
	3	4.2
	4	4.2
W1	1	6.5
	2	6.2
	3	6.3
	4	6.3
W2	1	6.5
	2	6.7
	3	6.9
	4	6.5
W3	1	6.5
	2	6.8
	3	6.6
	4	6.6
W4	1	6.8
	2	6.5
	3	6.6
	4	6.6

**Table 8:** Compressive strength of concrete with the addition of rubber granulates.

Sample	Subsample	Compressive Strength [MPa]
W0	1	12.1
	2	12.6
	3	12.5
	4	12.6
W1	1	21.0
	2	21.0
	3	20.8
	4	21.2
W2	1	21.6
	2	21.5
	3	21.6
	4	21.5
W3	1	21.0
	2	21.2
	3	21.2
	4	21.2
W4	1	21.0
	2	21.3
	3	21.1
	4	21.0

## Data Availability

Not applicable.
